# NF-kappaB Signaling Pathways in Neurological Inflammation: A Mini Review

**DOI:** 10.3389/fnmol.2015.00077

**Published:** 2015-12-18

**Authors:** Ruey-Horng Shih, Chen-Yu Wang, Chuen-Mao Yang

**Affiliations:** ^1^Institute of Neuroscience, National Chengchi UniversityTaipei, Taiwan; ^2^Department of Physiology and Pharmacology and Health Aging Research Center, College of Medicine, Chang Gung UniversityTao-Yuan, Taiwan

**Keywords:** NF-kappaB, neuroinflammation, neuroprotection, adhesion molecules, proinflammatory transcription factors

## Abstract

The NF-κB (nuclear factor κ-light-chain-enhancer of activated B cells) transcription factor family is a pleiotropic regulator of many cellular signaling pathways, providing a mechanism for the cells in response to a wide variety of stimuli linking to inflammation. The stimulated cells will be regulated by not only the canonical but also non-canonical NF-κB pathways. To initiate both of these pathways, IκB-degradation triggers NF-κB release and the nuclear translocated-heterodimer (or homodimer) can associate with the κB sites of promoter to regulate the gene transcriptions. NF-κB ubiquitously expresses in neurons and the constitutive NF-κB activation is associated with processing of neuronal information. NF-κB can regulate the transcription of genes such as chemokines, cytokines, proinflammatory enzymes, adhesion molecules, proinflammatory transcription factors, and other factors to modulate the neuronal survival. In neuronal insult, NF-κB constitutively active in neuron cell bodies can protect neurons against different injuries and regulate the neuronal inflammatory reactions. Besides neurons, NF-κB transcription factors are abundant in glial cells and cerebral blood vessels and the diverse functions of NF-κB also regulate the inflammatory reaction around the neuronal environment. NF-κB transcription factors are abundant in the brain and exhibit diverse functions. Several central nerve system (CNS) diseases are linked to NF-κB activated by inflammatory mediators. The RelA and c-Rel expression produce opposite effects on neuronal survival. Importantly, c-Rel expression in CNS plays a critical role in anti-apoptosis and reduces the age-related behaviors. Moreover, the different subunits of NF-κB dimer formation can modulate the neuroninflammation, neuronal protection, or neurotoxicity. The diverse functions of NF-κB depend on the subunits of the NF-κB dimer-formation which enable us to develop a therapeutic approach to neuroinflammation based on a new concept of inflammation as a strategic tool in neuronal cells. However, the detail role of NF-κB in neuroinflammation, remains to be clarified. In the present article, we provide an updated review of the current state of our knowledge about relationship between NF-κB and neuroinflammation.

## NF-κB Family Members and Disease Control

NF-κB exerts effects on almost all cell types in the body, playing an important function in inflammation, immune responses, cell cycle, and cell survival (Sen and Baltimore, 1986; Li and Verma, 2002; Kaltschmidt et al., 2005; Mattson, 2005; Ledoux and Perkins, 2014). NF-κB has been recognized as a member of Rel family of transcription factors. In mammals, there are five different members to compose the NF-κB family: p65 (RelA), RelB, c-Rel, p50/p105 (NF-κB1), and p52/p100 (NF-κB2) which have the similar amino acid sequence, the RHD (Rel homology domain, over approximate 300 amino acids) of these proteins (Chen and Greene, 2004). The activated NF-κB subunits will assemble to form the homo-or hetero-dimerized transcription factor complexes displaying the DNA-binding ability and transactivation potentials. The most widely studied form of NF-κB is a heterodimer of the p50 and p65 subunits and is a potent activator of gene transcription (Schmitz and Baeuerle, 1991). NF-κB is activated by a wide variety of agents including viruses, bacterial toxins such as lipopolysaccharide (LPS), UV light, oxidative stresses such as free radicals and cigarette smoke, inflammatory stimuli, cytokines, carcinogens, tumor promoters, and various mitogens (Baeuerle and Henkel, 1994; Baldwin, 1996). NF-κB regulates the expression of almost 500 different genes, including enzymes [e.g., cyclooxygenase (COX)-2, 5-lipoxygenase (LOX), and inducible NO synthase (iNOS)], cytokines [such as interleukin (IL)-1, IL-6, IL-8, chemokines, and tumor necrosis factor (TNF)], adhesion molecules, cell cycle regulatory molecules, and angiogenic factors (Duh et al., 1989; Kaltschmidt et al., 1993; Ahn and Aggarwal, 2005; Gupta et al., 2010a,b). The activation of NF-κB, especially the constitutively activated NF-κB in chronic inflammatory patients, has been found the critical linkage with a wide variety of human diseases, including asthma, atherosclerosis, AIDS, Alzheimer’s disease (AD), Parkinson’s disease (PD), rheumatoid arthritis, cancer, diabetes, and osteoporosis which belong to autoimmune/inflammatory diseases (Vallabhapurapu and Karin, 2009; Gupta et al., 2010b). The opposite, several native or artificial agents such as Th2 cytokines (IL-4, IL-13, and IL-10), interferons, endocrine hormones (LH, HCG, MSH, and GH), phytochemicals, corticosteroids, and immunosuppressive drugs, are known to block the specific signaling transductions and suppress NF-κB activation (Ahn and Aggarwal, 2005). Therefore, regulation and dysregulation of NF-κB play a key role in diseases control.

## The Canonical and Non-Canonical NF-κB Signaling Pathways

**FIGURE 1:**
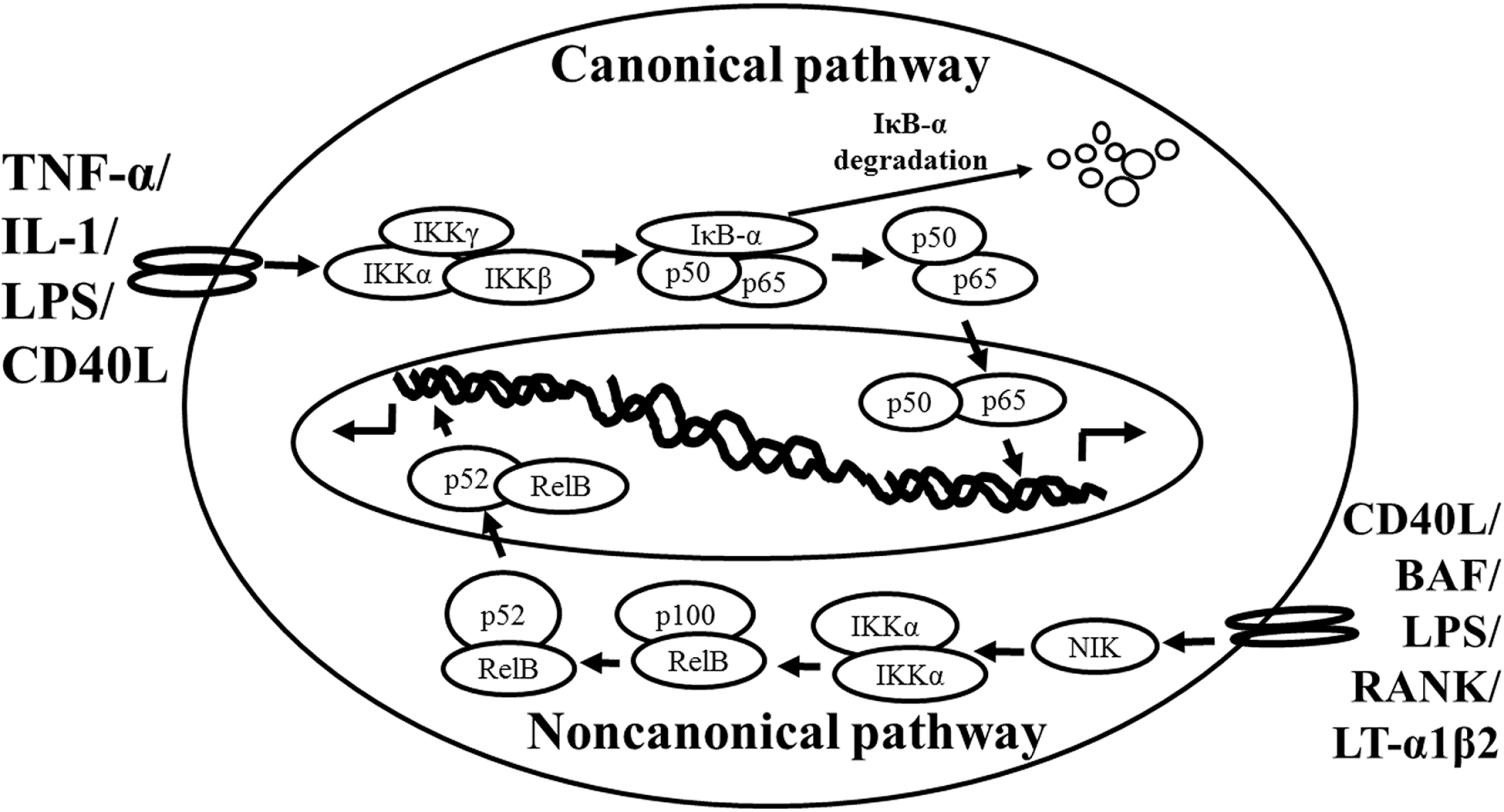
**Schematic representation of the canonical and non-canonical nuclear factor (NF)-κB activation pathways**. The canonical NF-κB pathway (upper) can be activated by a wide range of various stimuli, including tumor necrosis factor (TNF)-α, interlukin (IL)-1, lipopolysaccharide (LPS), and Toll-like receptors ligand (such as CD40L). Initiation of the canonical pathway via Toll-like receptor or cytokine receptor signaling depends on the inhibitor of κB kinase (IKK) complex, which is composed of the kinases IKKα and IKKβ, and the regulatory subunit IKKγ (NEMO). Activated IKK phosphorylates the inhibitory subunit IκBα leading to its degradation. The released NF-κB dimers (p50-p65) translocate to the nucleus and bind to κB site of chromosome to induce transcription of NF-κB targeted genes. The non-canonical pathway (lower) is activated by specific stimuli including B cell activating factor (BAF) belonging to the TNF family receptor, LPS, lymphotoxin (LT) α1β2, receptor activator of NF-κB (RANK), and CD40L. NF-κB inducing kinase (NIK) is stabilized. When stimulated, NIK is activated and recruits IKKα to the p100 complex to phosphorylate p100, leading to p100 ubiquitination. P52, the processing product of p100, generates the activated p52/RelB NF-κB complex, which is able to translocate to the nucleus and induce the downstream gene expressions.

Based on the previous studies, NF-κB is activated via two distinct kinase-dependent pathways, the classical/canonical NF-κB pathway and the alternative/non-canonical NF-κB pathway. The most extensively studied NF-κB activation pathway is the canonical pathway (**Figure 1**, modified from Noort et al., 2015), which can be mediated through activation of a variety of cell surface receptors, including IL-1 receptor, Toll-like receptors (TLRs), and TNF receptor, in response to pro-inflammatory mediators like IL-1, LPS, and TNF, as well as via triggering of the T-cell receptor or B-cell receptor. The inactive NF-κB resides in the cytoplasm and associates or links with the natural biological inhibitor IκB. The NF-κB function and nuclear translocation ability are sequestered in the cytoplasm and nuclear compartments, respectively (Verma et al., 1995; Baeuerle and Baltimore, 1996). The IκB family members include IκBα, IκBβ, p105/IκBγ (precursor of p50), p100 (precursor of p52), and IκBε (Li and Nabel, 1997; Whiteside et al., 1997). Each shares a series of ankyrin repeats which sequester NF-κB in the cytosol by masking its nuclear localization signal (NLS) and also prevents NF-κB from binding to DNA by masking its DNA binding domain. Treatment of cells with various stimuli activates IκB kinase complex, for example, leading to the phosphorylation of serines 32 and 36 of IκBα or serines 19 and 23 of IκBβ (DiDonato et al., 1997; Mercurio et al., 1997; Regnier et al., 1997; Zandi et al., 1997). These phosphorylation events target IκB for ubiquitin-dependent degradation through the 26S proteasome complex, resulting in the release and nuclear translocation of NF-κB (Finco and Baldwin, 1995; Thanos and Maniatis, 1995). Briefly, NF-κB is expressed ubiquitously in the cytoplasm of almost all cell types. The activated NF-κB will translocate from cytoplasm to nucleus and the NF-κB-dimer can bind to the κB site of promoter. In this classical pathway, inhibitor of κB kinase (IKK)β is required for NF-κB activation (Tak et al., 2001), whereas IKKα is redundant (Vallabhapurapu and Karin, 2009). However, the canonical NF-κB pathway is essential for both acute and chronic inflammatory responses. Moreover, this pathway is implicated in cell proliferation and survival, demonstrated by constitutively active NF-κB signaling in many tissues (Ben-Neriah and Karin, 2011).

The non-canonical NF-κB pathway (**Figure 1**), can be triggered by the activation of members of the TNF-receptor superfamily including B cell activating factor (BAF), belonging to the TNF family receptor, CD40, lymphotoxin β (LTβ) receptor, and receptor activator of NF-κB (RANK). Of note, these receptors trigger not only the non-canonical NF-κB pathway, but also the canonical pathway, simultaneously. The non-canonical NF-κB pathway is strictly dependent on IKKα homodimers and unlike the canonical pathway, the IKKβ or IKKγ is not involved in the IκB phosphorylation (Sun, 2012). To regulate the non-canonical pathway, expression of NF-κB inducing kinase (NIK) plays a role as the most important kinase. In the steady state, TNF receptor-associated factor (TRAF)3 mediates recruitment of NIK to TRAF2, which leads to NIK ubiquitination and continuous degradation. Consequently, endogenous levels of NIK are very low and the NF-κB complex is retained in the cytoplasm and kept inactive. Upon activation of the non-canonical NF-κB pathway, TRAF2 induces proteolysis of TRAF3. Degradation of TRAF3 prevents targeting of newly synthesized NIK, resulting in NIK release and accumulation. Subsequently, NIK induces p100 phosphorylation by IKKα homodimers and partial degradation to release p52. Next, mainly p52-RelB heterodimers translocate to the nucleus, leading to transcription of target genes. Whereas canonical NF-κB activation is rapid and independent of protein synthesis, non-canonical NF-κB activation requires NIK synthesis and accumulation. Consequently, the kinetics of this pathway are considerably slower (Vallabhapurapu and Karin, 2009; Sun, 2012).

There are cross-talks between these two pathways. IKKα has, for instance, been described to also have nuclear functions and serve as a regulator of canonical NF-κB-dependent gene expression through control of promoter-associated histone phosphorylation exposed to cytokines (Anest et al., 2003; Yamamoto et al., 2003). It has been demonstrated that the activated canonical pathway not only initiated the signal transduction of NF-κB but also suppressed basal non-canonical signaling in immune cells (Gray et al., 2014). Interestingly, under certain circumstances and other stimuli (including TNF) can also activate non-canonical NF-κB signaling in specific cell types (Zhang et al., 2014), and IKKα is critical for interferon-α production induced by TLR 7 and 9 (Hoshino et al., 2006).

## The NF-κB Family Members in the Brain Location

The expression of NF-κB transcription factors is abundant in the brain. The basal levels of NF-κB expression have been identified in the brain where their amounts are higher than those of peripheral tissues. Several lines of evidence indicate that constitutively activated NF-κB is found in glutamatergic neurons of the central nervous system (CNS), such as cerebral cortex (layers 2, 4, and 5) and hippocampus (granule cells and pyramidal neurons of CA1 and CA3; Kaltschmidt et al., 1993, 1994, 1995). A number of studies also show constitutive NF-κB activity in various rodent brain regions such as amygdala, cerebral cortex, cerebellum, hippocampus, hypothalamus, and olfactory lobes (Schmidt-Ullrich et al., 1996). Among members of NF-κB, all of the complexes of c-Rel/p65, p50/p65 heterodimer, and p50 homodimers are detected in the developing rat brain (Bakalkin et al., 1993). While to analyze the distribution of NF-κB, the released p65 and p50 NF-κB subunits are abundantly expressed in neurons. Moreover, p50/p65 heterodimers are located in the cell nucleus and exhibit constitutive activity in the adult brain (Kaltschmidt et al., 2005; Meffert and Baltimore, 2005). In the developed rodent brain, the p50/p65 heterodimeric variant of NF-κB is converted to the major κB-binding complex (Schmidt-Ullrich et al., 1996; Meffert et al., 2003). It is important for the neuronal physiological characteristics, for example, constitutive NF-κB activity in glutamatergic neurons of the hippocampus and cerebral cortex can be suppressed by N-methyl-D-aspartate, and to a lesser extent AMPA, glutamate receptor antagonists, as well as L-type Ca^2+^ channel blockers (Lilienbaum and Israel, 2003; Meffert et al., 2003). These studies suggest that constitutive NF-κB activity is modulated by physiological basal synaptic transmission. However, inducible NF-κB is detected in synapses, glutamatergic stimulation activates retrograde transport of p65 protein from synapses to the cell nucleus (Kaltschmidt et al., 1993; Meberg et al., 1996; Meffert et al., 2003). Thus, NF-κB is involved in translation of short-lasting synaptic signals to persistent changes in gene expression (Wellmann et al., 2001; Meffert et al., 2003). The activated IKK ant it’s product, phosphorylated IκBα, were detected within the axon initial segment, the site where action potentials are generated (Schultz et al., 2006), suggesting that constitutive NF-κB activation is involved in the processing of neuronal information.

## NF-κB and Neuroinflammatory Mediators

At the molecular level, inflammation is regulated by numerous molecules and factors, including adhesion molecules [intercellular adhesion molecule (ICAM-1), vascular cell adhesion molecule (VCAM)-1, endothelial-leukocyte adhesion molecule (ELAM)-1], chemokines (such as monocyte chemoattractant protein 1, IL-8), cytokines (IL-1, IL-2, IL-6, IL-12, TNF-α, TNF-β), signal transducer and activator of transcription (STAT)-3, proinflammatory enzymes [COX-2, 5-LOX, 12-LOX, matrix metalloproteinases (MMPs), prostate-specific antigen (PSA), C-reactive protein], vascular endothelial growth factor (VEGF), and proinflammatory transcription factors NF-κB (Aggarwal, 2004). Among these mediators, NF-κB is the central regulator of inflammation (Lukiw and Bazan, 1998; Aggarwal, 2004; Ahn and Aggarwal, 2005). For example: IL-1β treatment can induce COX-2 expression in canine tracheal smooth muscle cells (Yang et al., 2002) and ICAM-1 expression in human rheumatoid arthritis synovial fibroblasts (Yang et al., 2010), respectively. LPS, to mimic the bacterial infection, and endothelin-1 also can induce COX-2 and PGE_2_ expression in mouse brain microvascular endothelial (bEnd.3) cells (Shih and Yang, 2010; Lin et al., 2013). TNF-α can induce ICAM-1 expression in osteoblast-like MC3T3-E1 cells (Tsai et al., 2014). All of these target proteins syntheses are mediated through NF-κB-dependent signaling pathway.

NF-κB has been shown to activate more than 500 genes, which are implicated in inflammation related responses (Gupta et al., 2010a,b). The NF-κB family is suggested to be the most extensively studied target in inflammation issue for its critical role (Chen and Greene, 2004; Lin and Karin, 2007). In neuroinflammation, NF-κB can be transiently activated by various stimuli, like acute alcohol exposure, which induces neuroinflammatory responses in mice (Yakovleva et al., 2011). The role of NF-κB is critical in the regulation of neuroinflammation-associated disease pathogenesis (Niranjan, 2013).

## NF-κB: A Neuroprotective Role or a Neurotoxic Role

In the CNS, NF-κB transcription factors are key players in a number of physiological processes such as neurogenesis (Koo et al., 2010), neuritogenesis (Rolls et al., 2007), and synaptic plasticity which related to learning and memory (Levenson et al., 2004; O’Riordan et al., 2006; Ahn et al., 2008). A number of studies also provide evidence that activation of NF-κB protects neurons against the different injuries such as excitotoxicity (Mattson, 2005), and oxidative stress (Sarnico et al., 2009b), as well as amyloid β peptide toxicity (Barger et al., 1995; Kaltschmidt et al., 1997) and exerts as a cellular defense program. Apoptotic cortical neurons have been observed to be rescued by overexpression of p65, while enhanced damage by IκB super-repressor or dominant negative NF-κB-inducing kinase (NIK; Bhakar et al., 2002). NF-κB is constitutively active, and involved in neuronal injury as well as neuroprotection in neuron cell bodies, however, NF-κB is present in a latent form at the synapse. Only when NF-κB is activated, it can be transported to the neuron cell nucleus (Yakovleva et al., 2011).

Besides neurons, the roles of NF-κB in astroglia/microglia have been studied in relation to brain injury (O’Neill and Kaltschmidt, 1997; Block et al., 2007; Kaltschmidt and Kaltschmidt, 2009). Briefly, NF-κB is present in a latent form in glia of naive animals (Schmidt-Ullrich et al., 1996; Bhakar et al., 2002). NF-κB may be activated under pathological conditions such as exposure to HIV-1 Tat or amyloid β peptide (Aβ) leading to the production of nitric oxide (Akama et al., 1998; El-Hage et al., 2008). It has been shown that glia responses to injury triggered by endogenous ligands for TLR and TLR signaling are mediated through the NF-κB (Akira and Takeda, 2004). Moreover, inhibition of astroglial NF-κB signaling leads to reduced chemokine expression and leukocyte entry into the injured CNS (Brambilla et al., 2005; Khorooshi et al., 2008). NF-κB has been shown to play the regulatory role of astrocytes on immune and inflammatory responses (Farina et al., 2007). Microgliosis is a common pathologically neurodegenerative disorder. Microglial activation of NF-κB plays a central role associated with the release reactive oxygen species and proinflammatory cytokines (such as IL-1β, interferon-γ, and TNF-α) that can cause secondary neurotoxicity (Kaltschmidt et al., 1993; Block et al., 2007). Briefly, in glia, NF-κB is inducible and regulates inflammatory processes that exacerbate inflammation-induced neurodegeneration (Yakovleva et al., 2011). NF-κB has been also demonstrated as a major signal transducer affecting cellular permeability, endocytosis, and intracellular trafficking at the level of the blood–brain barrier (Stone et al., 2011). Activation of NF-κB signaling by LPS has been shown to induce inflammatory target protein COX-2 and PGE_2_ production leading to cerebral vascular inflammation (Pan et al., 2010; Shih and Yang, 2010). All of above studies show that NF-κB transcription factors are abundant in the brain where they have diverse functions among neurons, glia, and cerebral blood vessels.

## Effects of NF-κB on Inflammatory-Associated with Pain

Constitutive activation of NF-κB is detected mostly in glutamatergic neurons. NF-κB in glia has a lower basal activity and is highly inducible, which plays a crucial role in brain inflammation (Kaltschmidt and Kaltschmidt, 2009). A role of glial NF-κB in pain research has attracted more attention. Pain signaling can arise from the activation of specific high-threshold PNS neurons (nociceptors) and could serve as a sensing mechanism to prevent further injury. In clinic, pain signaling can arise not only from damage to the nervous system (neuropathic pain), but also from chronic inflammation (inflammatory pain). Interestingly, an impairment of acute and inflammatory nociception has been revealed in p50^-/-^ mice in a previous study (Niederberger et al., 2007). Moreover, inhibition of astroglial NF-κB can reduce inflammation and therefore improve functional recovery after spinal cord injury (Brambilla et al., 2005). All of these data suggests that NF-κB plays a crucial role on inflammatory pain in CNS.

## Different NF-κB Complexes Differentially Regulate Neuronal Survival in Brain Damage: p50/RelA vs. p50/c-Rel

In recent years, NF-κB dysregulation has been shown to link to neurodegenerative mechanisms that occur in brain during trauma or ischemia (Bethea et al., 1998; Schneider et al., 1999), as well as in the brain of patients suffered by PD (Hunot et al., 1997; Ghosh et al., 2007) or AD (Boissiere et al., 1997; Kaltschmidt et al., 1997; Lukiw and Bazan, 1998). These CNS diseases are associated with neuroinflammatory mediators. More evidence has shown that the neuronal response to external stimuli relies on a differential activation of NF-κB dimers. RelA or c-Rel expression produces opposite effects on neuron survival (Pizzi et al., 2002, 2005b; Sarnico et al., 2009b).

Among the members of NF-κB, the RelA subunit, composing the activated p50/RelA dimer, and its post-transcriptional modifications play a pivotal role in the onset of neurodegenerative processes triggered by ischemic insults (Inta et al., 2006; Sarnico et al., 2009a,b) as well as glutamate (Pizzi et al., 2002) or Aβ toxicity (Pizzi et al., 2005b; Inta et al., 2006; Lanzillotta et al., 2010). In ischemic stroke, activated RelA induces the expression of the 1B isoform of the divalent metal transporter-1(1B/DMT1) which can exert as an upstream response for iron accumulation and contributing to neuronal cell death after injury (Ingrassia et al., 2012). Notably, RelA is demonstrated as a most contributing subunit in degenerative changes associated with senescence in a mice model (Tilstra et al., 2012).

RelA has been demonstrated to contributing to neuronal cell death, while the overexpression of c-Rel factor can limit the cell death. The c-Rel factor is reduced in neurons exposed to oxygen–glucose deprivation (OGD), interestingly, the overexpression of c-Rel prevents neuronal loss in cortical neurons exposed to OGD. This protective effect involves in increasing the transcription of Bcl-xL gene (Pizzi et al., 2009; Sarnico et al., 2009a,b). Similarly, knocking down c-Rel expression exacerbated neuronal susceptibility to OGD-mediated damage (Pizzi et al., 2009). Further, knocking out c-Rel expression appeared insensitive to neuroprotective activity of leptin, a c-Rel inducer capable to limit cortical damage in wild-type mice and mice brain ischemia (Valerio et al., 2006, 2009). Therefore, the c-Rel subunit within activated NF-κB dimers also counteracts the ischemic injury acting as an innate mechanism of neuroprotection (Sarnico et al., 2009a,b). In addition, overexpression of c-Rel in cultured neurons promotes anti-apoptotic effects by inducing the transcription of manganese superoxide dismutase (MnSOD; Chen et al., 2000; Bernard et al., 2001; Pizzi et al., 2005a). On the viewpoint of disease events, the deficiency of c-Rel induces an age-related behavioral Parkinsonism in mice, with degeneration of nigral dopaminergic (DA) neurons and development of a PD-like neuropathology (Baiguera et al., 2012). Recent evidence has shown that activation of NF-κB drives the systemic and brain aging processes in mice (Adler et al., 2007; Zhang et al., 2013). In brain ischemic tissue of mice subjected to permanent middle cerebral artery occlusion (MCAO) and in primary cortical neurons exposed to OGD, NF-κB followed a pattern of increasing p50/RelA dimmer (Crack et al., 2006; Inta et al., 2006) and decreasing c-Rel-containing dimmers (Sarnico et al., 2009b). Inhibition of c-Rel-containing dimers and activation of p50/RelA are key events in the pathogenesis of brain injury. These data strongly suggested that NF-κB transcription factors have diverse functions that depend on the composition of the NF-κB complex (Lanzillotta et al., 2015).

## Conclusion

**FIGURE 2:**
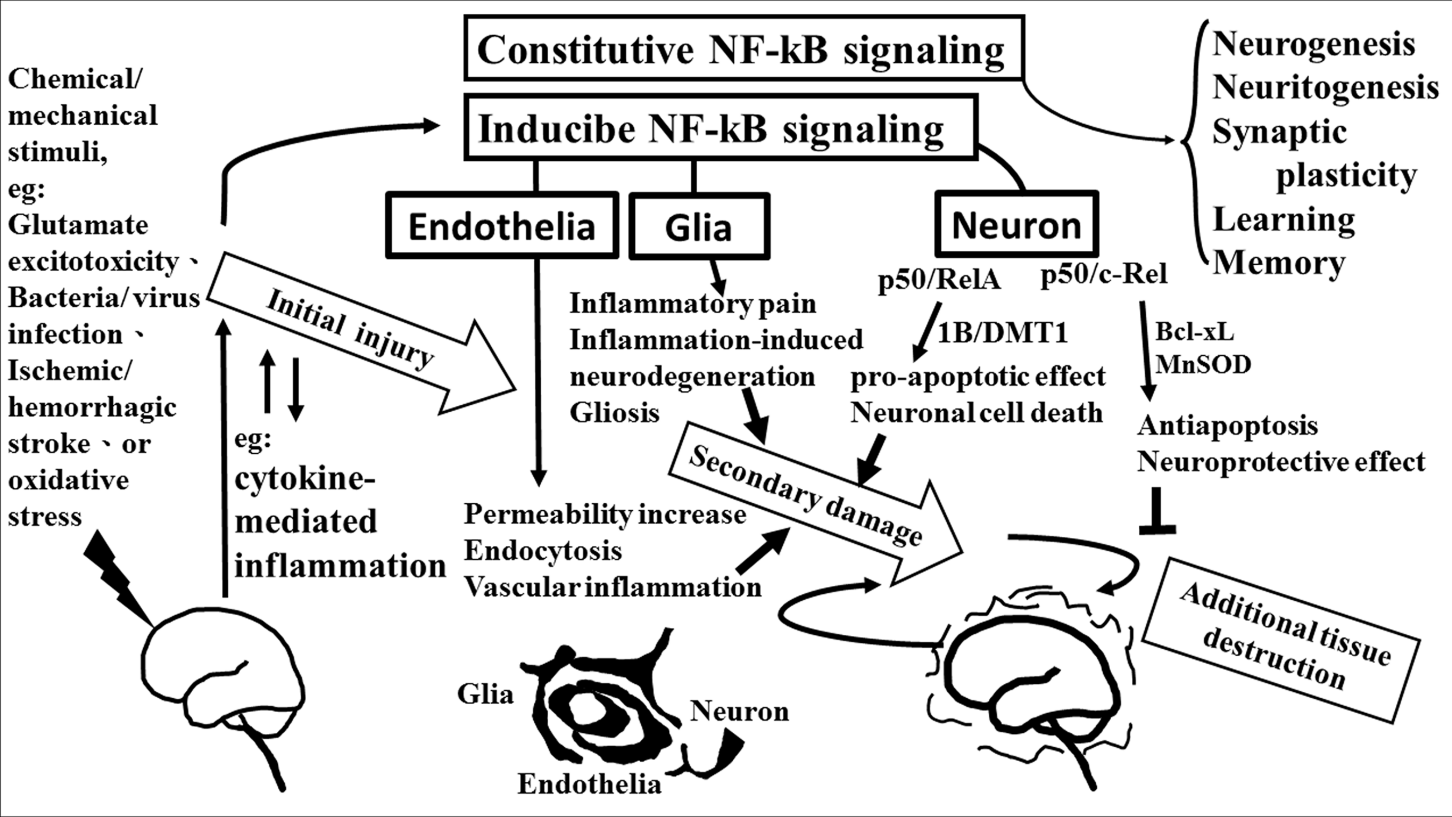
**The role of NF-κB in neurological damage**. Chemical/mechanical stimulation (such as glutamate excitotoxicity, bacteria/virus infection, ischemic/hemorrhagic stroke, or oxidative stress) to the brain/spinal cord tissue results in initial injury, including glutamate neuron-excitotoxicity, and cytokine-mediated inflammation which increase oxidative stress linking to neuroinflammatory response. NF-κB transcription factors are abundant in the brain where they have diverse functions between neuron, glia, and cerebral blood vessels. Constitutive NF-κB transduction factors are responsible for neurogenesis, neuritogenesis, synaptic plasticity, learning, and memory. Either glial or endothelial inducible NF-κB activation was implicated in neuroinflammation-associated pathogenesis related to secondary neuronal damage, while p50/RelA and p50/c-Rel subunit within activated NF-κB dimers play different roles on neuronal pathogenesis in neuron. The p50/RelA enhances damage by inducing the expression of the 1B isoform of the divalent metal transporter-1(1B/DMT1), p50/c-Rel protects against the damage by increasing the transcription of gene of Bcl-Xl or MnSOD. NF-κB transcription factors have diverse functions that depend on the composition of the NF-κB complex and cell types.

The role of NF-κB is critical in the regulation of neuroinflammation-associated disease pathogenesis. NF-κB transcription factors are abundant and constitutive activation in brain where they have diverse functions among neurons, glia, and cerebral blood vessels. These functional diversions are dependent on the recruitment of components of the NF-κB dimer formation. Especially, c-Rel containing NF-κB dimers can induce the Bcl-xL and MnSOD expression and exert as anti-apoptotic effects while the pro-apoptotic effect elicited by NF-κB p50/RelA dimer. The imbalance of NF-κB dimer formation between RelA and c-Rel might result in the pathological process in certain neurons. The roles of NF-κB in neurological damage have been illustrated in **Figure 2**. The detail signal transduction pathways in different compositions of the NF-κB complex remain to be clarified. Obviously much more work is required to elucidate the role of NF-κB in the neuroinflammatory signaling pathways, which in turn will enable us to devise a therapeutic approach to neuroinflammation based on a new concept of inflammation as a strategic tool by which inflammatory neuronal cells can be made more susceptible to drugs than normal cells. By understanding the signal transduction pathways mediating the induction of NF-κB in neuronal cells, it may be possible to manipulate these diseases for therapeutic gain.

## Conflict of Interest Statement

The authors declare that the research was conducted in the absence of any commercial or financial relationships that could be construed as a potential conflict of interest.
